# The effect of habitat fragmentation on the bee visitor assemblages of three Australian tropical rainforest tree species

**DOI:** 10.1002/ece3.4339

**Published:** 2018-07-22

**Authors:** Tobias J. Smith, Margaret M. Mayfield

**Affiliations:** ^1^ School of Biological Sciences The University of Queensland St Lucia Qld Australia

**Keywords:** Apoidea, functional traits, *Tetragonula*, tropical forest

## Abstract

Tropical forest loss and fragmentation can change bee community dynamics and potentially interrupt plant–pollinator relationships. While bee community responses to forest fragmentation have been investigated in a number of tropical regions, no studies have focused on this topic in Australia. In this study, we examine taxonomic and functional diversity of bees visiting flowers of three tree species across small and large rainforest fragments in Australian tropical landscapes. We found lower taxonomic diversity of bees visiting flowers of trees in small rainforest fragments compared with large forest fragments and show that bee species in small fragments were subsets of species in larger fragments. Bees visiting trees in small fragments also had higher mean body sizes than those in larger fragments, suggesting that small‐sized bees may be less likely to persist in small fragments. Lastly, we found reductions in the abundance of eusocial stingless bees visiting flowers in small fragments compared to large fragments. These results suggest that pollinator visits to native trees living in small tropical forest remnants may be reduced, which may in turn impact on a range of processes, potentially including forest regeneration and diversity maintenance in small forest remnants in Australian tropical countryside landscapes.

## 1 INTRODUCTION

The fragmentation of terrestrial ecosystems is one of the biggest contributors to biodiversity loss on the planet (Dirzo & Raven, 2003; Hanski, 1998; Harrison & Bruna, 1999; Millennium Ecosystem Assessment, 2005), and the negative consequences have been documented in numerous plant and animal groups (e.g., Arroyo‐Rodriguez & Dias, 2010; Cushman, 2006; Didham, Ghazoul, Stork, & Davis, 1996; Turner, 1996). In addition to effects on individual species and groups, habitat fragmentation can affect core ecosystem processes (Valladares, Salvo, & Cagnolo, 2006), further endangering species within fragmented ecosystems.

In the tropics, high rates of deforestation (Wright & Muller‐Landau, 2006) have resulted in the fragmentation of most forests (Laurance, 1999). Tropical rainforests are the most species‐rich terrestrial ecosystems on the planet (Lewis, 2009), and understanding the threats to their diversity‐maintaining processes due to habitat fragmentation is essential not only for biodiversity conservation, but also for the prosperity of the human communities that rely on these forests for a wide range of ecosystem services. One mutualism‐based ecosystem process that provides a direct service to tropical human communities is biotic pollination (Blanche, Ludwig, & Cunningham, 2006; Garibaldi et al., 2016; Ricketts, 2004; Ricketts et al., 2008). Mutualistic plant–animal relationships such as biotic pollination are particularly vulnerable to habitat loss and fragmentation because of the typically high levels of connectivity of their interaction networks (Aizen & Feinsinger, 1994; Fortuna & Bascompte, 2006; Harris & Johnson, 2004; Rathcke & Jules, 1993). Disruptions to these processes can have deleterious consequences for plant communities (Dauber et al., 2010; Sekercioglu, Daily, & Ehrlich, 2004) and may ultimately lead to further decay of key ecological processes (Koh et al., 2004; Lever, van Nes, & Bascompte, 2014; Pauw, 2007; Ramos‐Jiliberto et al., 2009; Rathcke & Jules, 1993).

Bees (Hymenoptera: Apoidea) are the most important pollinator group globally. Empirical studies on the effects of habitat fragmentation on bees are historically uncommon (Cane, 2001), and even with a rise in interest in this topic over recent years, most studies that have been conducted focus on the neotropics (e.g., Aizen & Feinsinger, 1994; Brosi, 2009; Brosi, Daily, Shih, Oviedo, & Duran, 2008; Calvillo, Ramirez, Parra‐Tabla, & Navarro, 2010; Ferreira et al., 2015; Powell & Powell, 1987; Tonhasca Jr, Blackmer, & Albuquerque, 2002). While these studies have had mixed results, including both negative (e.g., Aizen & Feinsinger, 1994; Calvillo et al., 2010) and neutral (e.g., Tonhasca Jr et al., 2002) effects of fragmentation on bee diversity, they all tend to show that different bee groups respond differently to fragmentation, probably as a result of distinct nesting and foraging traits among species (Brosi et al., 2008).

One tropical region where work on the ecology of bee communities is still in its infancy is the Australian wet tropics. The Australian wet tropics bioregion of northern Queensland is a diversity hotspot for a range animals and plants, despite covering 0.1% of the continent's land surface (Goosem, Morgan, & Kemp, 1999; Kikkawa, 2008). Although considered to have a “depauperate” bee fauna relative to elsewhere in Australia (Michener, 1965), approximately 11% of Australia's known bee species occur in the Australian wet tropics (Atlas of Living Australia, 2018; Australian Faunal Directory, 2018). Studies of bee diversity across fragmented landscapes have been conducted elsewhere in the tropics; however, no such studies have occurred in Australian tropical landscapes. The wet tropics studies that have reported on bee communities in rainforest have focused on pollinators or floral visitors to specific focal rainforest plant species (e.g., Boulter, Kitchling, Howlett, & Goodall, 2005; Gross & Mackay, 1998; House, 1989; Kitching, Boulter, Howlett, & Goodall, 2007), often with little taxonomic precision, rather than investigating bee communities across whole landscapes (but see Blanche et al., 2006).

It has become increasingly clear that in order to make more direct links to ecosystem processes, studies need to investigate patterns of functional trait diversity as well as taxonomic diversity (Cadotte, Carscadden, & Mirotchnick, 2011; Didham et al., 1996; Flynn et al., 2009). All organisms have measureable functional traits that are ecologically important and that have the potential to affect performance and fitness (Cadotte et al., 2011). Although functional traits have been best studied in plants (e.g., Diaz et al., 2004; Pérez‐Harguindeguy et al., 2013), studies of insect communities that incorporate functional trait diversity are becoming more common (e.g., Horgan, 2008; Taillefer & Wheeler, 2012). We are only aware of one study of tropical bee communities, however, that investigates any functional traits (see Kambach, Guerra, Beck, Hensen, & Schleuning, 2013). In many regions of the world, much of the remaining forest in tropical agricultural landscapes is fragmented (Arroyo‐Rodriguez, Pineda, Escobar, & Benitez‐Malvido, 2009), and as such, it is imperative that studies focusing on functional diversity are undertaken to more fully understand the long‐term persistence of these ecosystems.

Here, we use the floral visitation data involving bees and three tree species to assess the effects of habitat fragmentation in the Australian wet tropics, focusing on both the taxonomic and functional diversity of bee visitors. With increasing concern for the future of pollinators and pollination, it is essential to understand how processes such as habitat fragmentation affect both taxonomic and functional diversity of tropical bee communities. In this study, we ask two questions: (a) Does the richness, abundance, and composition of bees visiting three common floral host trees differ between small and large forest fragments in fragmented Australian tropical landscapes? and (b) In addition to any taxonomic variation, are there detectable functional differences in the bee assemblages found in large and small forest fragments visiting the same host tree species?

## 2 METHODOLOGY

### 2.1 Study location

**Figure 1:**
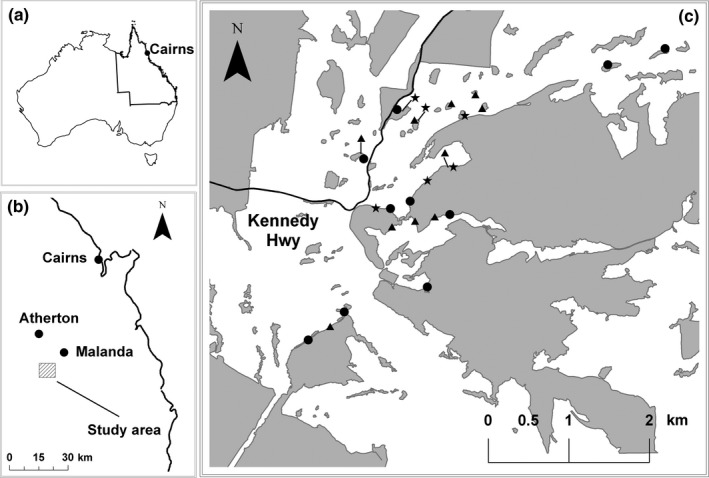
Map of the study region on the Atherton Tableland, north Queensland, Australia (a,b), showing focal tree sites across 14 fragments used in this study (c). Shaded areas represent forest patches. (▲) *Acronichia acidula*, (★) *Sarcopterix reticulata*, and (●) *Alphitonia petriei*

**Figure 2:**
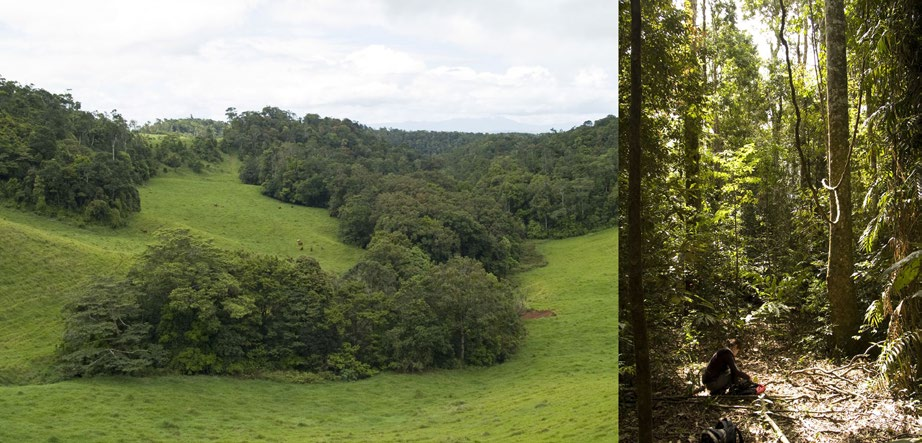
Photographs that typify our study landscapes in the Upper Barron region of the Atherton Tableland, Queensland. On left, remnant forest surrounded by a matrix of cattle pasture, and on right, typical structure of the forest within fragments in our landscape (photograph taken from within a forest gap)

This study took place in the Upper Barron region of the Atherton Tableland, Queensland (Figure 1). The Atherton Tableland was once extensively covered by rainforest, but after decades of land clearing from the late 1800s (Western & Goosem, 2004), the small amount of remaining forest is now highly fragmented. The study region now consists of a heterogeneous landscape primarily composed of cattle pasture and remnant forest fragments of various sizes (Figure 2).

### 2.2 Sites and species

We sampled floral bee visitor assemblages of three tree species: *Acronychia acidula* (Rutaceae), *Alphitonia petriei* (Rhamnaceae), and *Sarcopteryx reticulata* (Sapindaceae). We surveyed bees visiting flowers of *A. petriei* and *S. reticulata* in September 2010, which is in the middle of the region's dry season and peak flowering time for these species. *A. acidula* blooms in the wet season, and thus, our surveys of bees visiting this species’ flowers were conducted in January and February 2010. Twenty‐six individual trees (10× *A. acidula*, 10× *A. petriei*, and 6× *S. reticulata*) were identified among four large (>20 Ha) and ten small (<5 Ha) rainforest fragments across the study region (Figure 1c, Supporting Information Table S1). All trees were located on forest edges, where low hanging branches were more easily accessible. Within each species, we selected trees across the landscape that were of similar overall size, and on each, we netted all accessible branches between ground level and a height of 5 m. For small‐sized forest fragments, we netted only one individual tree of each species per fragment. In some large forest fragments, there were instances of multiple trees of a single species within a single fragment. In these cases, trees were separated by a minimum of 200 m. This repeated sampling within fragments was dealt with statistically and is explained in detail below.

*Acronychia acidula* grows well in disturbed areas, characteristically being found in rainforest regrowth (Hyland, Whiffin, Christophel, Gray, & Elick, 2002). Seed dispersal is by flying vertebrates (Sonter, Metcalfe, & Mayfield, 2011). No specific floral visitor information is available for the species or genus, although other Rutaceae species with similarly structured flowers are pollinated by insects (Armstrong, 1979).

*Alphitonia petriei* is a fast‐growing pioneer species (Sun, Dickson, & Bragg, 1995), often seen in our study region growing in regrowth rainforest, along forest edges, and as seedlings in abandoned pasture. Seed dispersal is by birds (Florentine & Westbrooke, 2004). A review of floral visitors to Australian flora lists species from the four major Australian bee families (Apidae, Colletidae, Halictidae, and Megachilidae) as floral visitors of *Alphitonia* (Armstrong, 1979).

*Sarcopteryx reticulata* is a rainforest understory tree (Hyland et al., 2002), which commonly occurs along forest edges in our study region. Seeds are dispersed by flying vertebrates (Sonter et al., 2011). While information on its pollinators is unavailable, other species in the genus are thought to be bee‐pollinated (Sonter et al., 2011).

### 2.3 Insect collections

Floral bee visitors were sampled once at each individual tree, using sweep netting around inflorescences on low‐hanging branches (<5 m above the ground). At each tree, three sweeps of 20 s each were conducted over an area of flowering branches, with 3–5 min between sweeps. For *S. reticulata*, which has a dense form, sweeping was conducted across the side of the tree. In the other two tree species, sweeps were conducted along the tops of flower‐bearing branches.

Flower visitor diversity at individual plants can vary at multiple temporal scales over a flowering season (Herrera, 2005; Price, Waser, Irwin, Campbell, & Brody, 2005; Valverde, Gómez, & Perfectti, 2016). As we sampled the visitors at each of our 26 individual trees only once, we tried to minimize weather‐related variation in the visitor assemblages by keeping weather conditions as consistent as possible across sampling days. Notably, all netting was undertaken between 10:00 a.m. and 3:00 p.m. in sunny warm conditions, and no netting was undertaken during periods of fog, rain, mist, or high winds (for daily maximum temperatures on sampling days, see Supporting Information Table S2).

Bees were identified to genus level using the keys of Michener (2007, 1965), and the resulting reference collection of morphospecies was sent to the Australian Museum for species‐level identifications (see Acknowledgements). Each species was scored on a range of functional traits. For each species in our study, we recorded the following: mean intertegular distance (ITD—distance between wing bases, based on measurements of as many pinned individual females as we had available for each species); nesting habit (cavity nesters, those that nest in abandoned insect bores in wood or in dry twigs and stems, and soil burrowers); and sociality (eusocial, semisocial, or solitary). Nesting habit and sociality were identified for each species using information sourced from Michener (1960, 1965, 2007). Past studies have identified all of these traits as important in determining the ecological role of bee species in communities (Moretti, de Bello, Roberts, & Potts, 2009; Williams et al., 2010).

### 2.4 Data analysis

As we sampled multiple tree species within some of the individual forest fragments, we included individual fragment ID (*n* = 14) as a random factor (nested within tree species and fragment size) in all of the following analyses on measures of taxonomic diversity. In addition to this, we also tested for spatial autocorrelation (latitude and longitude) among trees for each response variable. This was made using the RELATE function in Primer v6+ PERMANOVA (Primer‐E Ltd, 2008), which calculates Spearman's rank correlation coefficients (rho) between resemblance matrices nonparametrically and is analogous to a Mantel test (Clarke & Gorley, 2006). No spatial autocorrelation among sites was detected for any variable (Supporting Information Table S3). All analyses were performed in Primer v6+ PERMANOVA, and all data were square‐root‐transformed prior to analysis (unless specified below).

To identify any effect of tree species or fragment size on species richness or abundance, we used two univariate permutational analyses of variance (PERMANOVAs), type III sums of squares with 9999 permutations, using Euclidean‐based dissimilarity values. In this way, PERMANOVA performs the same as a traditional ANOVA, but calculates *p*‐values using permutations rather than using tabled *p*‐values and as such does not require data to be normally distributed (Anderson, 2005). For the multivariate analysis, we used Monte Carlo *p*‐values.

To determine whether there was an effect of tree species and fragment size on bee visitor community composition, we ran a multivariate permutational analysis of variance (PERMANOVA), type III sums of squares with 9,999 permutations, based on Bray–Curtis resemblance values, with two fixed factors, tree species and fragment size, and fragment ID as a random factor. We used Monte Carlo *p*‐values, *p*(mc) (Anderson, Gorley, & Clarke, 2008), as the maximum number of unique permutations was not adequate in some instances, due to the low number of replicate trees (*S. reticulata*). To assist in visualizing effects identified using PERMANOVA, we ran a principal coordinate analysis ordination, which plots samples (individual trees) against one another in two dimensions depending on the similarity of their bee community composition. We used similarity percentage analysis (SIMPER) to identify the level of influence of individual bee species on any overall community dissimilarity between large and small fragments identified by PERMANOVA.

We used univariate PERMANOVA, as above, to identify any effect of tree species and fragment size on individual bee species identified as having disproportionately large impacts on dissimilarity, as identified by SIMPER. In addition, we ran a univariate PERMANOVA to identify whether there was a relationship between fragment size and bee ITD (ITD of individuals, data pooled within fragment size, data not transformed), which was the only trait with enough variability of states among sites for meaningful comparison.

## 3 RESULTS

### 3.1 Taxonomic diversity

**Figure 3:**
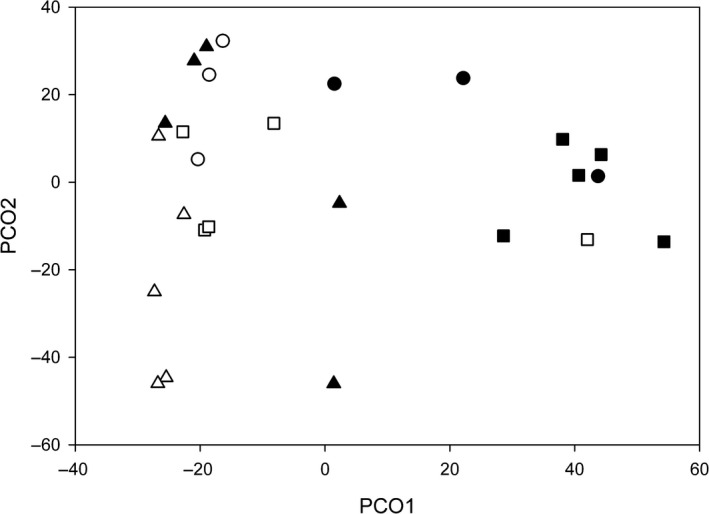
Principal coordinate analysis ordination (PCO) showing the variation in bee visitor assemblages among the different individual trees in this study. Points (individual trees) that are closer together on the plot are more similar in bee visitor composition. (▲) *Acronichia acidula*, (●) *Sarcopterix reticulata*, and (■) *Alphitonia petriei*. Individuals in black were in large fragments, while those in white were in small fragments

**Figure 4:**
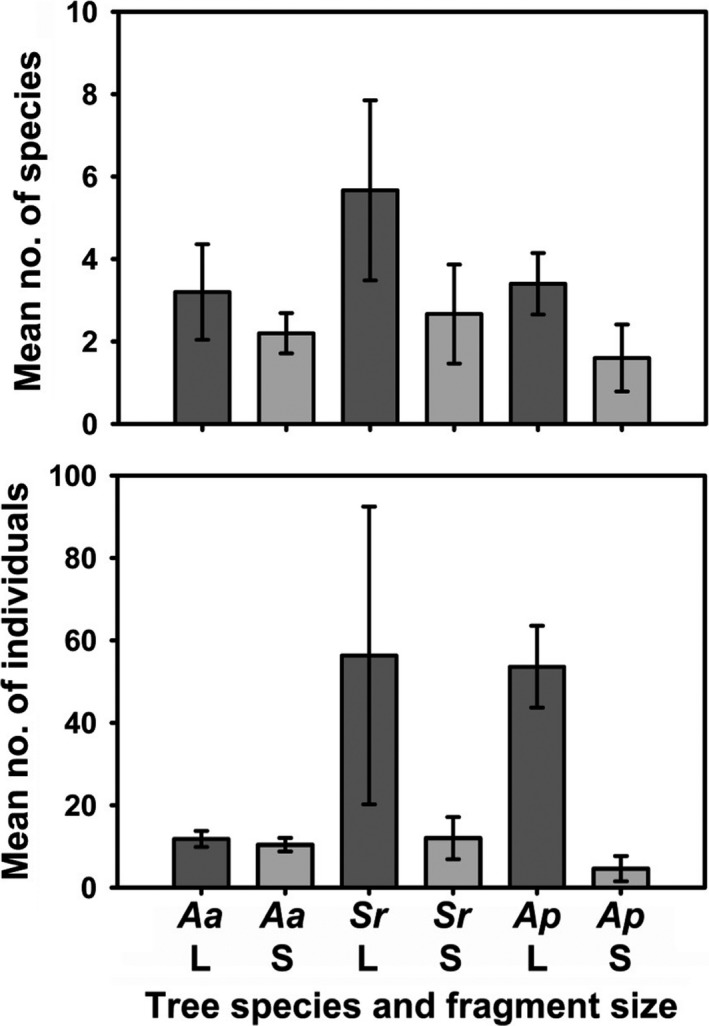
Mean number of bee species (top) and mean bee abundance (bottom) among the three tree species and two fragment sizes, +/− *SE*. *Aa *=* Acronichia acidula*;* Sr *= *Sarcopterix reticulata*;* Ap *= *Alphitonia petriei*; L = large fragments (dark gray); and S = small fragments (light gray)

Across the study, we collected 607 individual bees, from 18 species (Table 1). We found a significant effect of fragment size on community composition, overall species richness, and overall abundance (Figures 3 and 4; Table 2). For community composition, we also found a significant effect of tree species (Figure 3; Table 2). For both community composition and abundance, there was a significant interaction effect between fragment size and tree species (Table 2), demonstrating that while there was an overall effect of fragment size, the level of this effect varied among the tree species (Figures 3 and 4). Overall, the bee species found visiting trees in small fragments were a subset of the species found visiting trees in large fragments (Table 1). One bee species was found in small fragments only, while eight species were found in large fragments only (not in small fragments; Table 1). Individual fragment identity had no significant effect on community composition, total species richness, or total abundance (Table 2).

**Table 1:** List of individuals of each species collected in this study. Also included is the mean intertegular distance (ITD) of each species, as well as its nesting strategy and level of sociality. Nesting and sociality information was sourced from Michener (1960, 1965, 2007)

Family	Species	Habitat detected	Tree species visited	Mean IT (mm)	Nesting strategy	Level of sociality	Total collected
Apidae	*Apis mellifera*	Both	All	2.9	Cavity nests	Eusocial	114
	*Braunsapis simillima*	Large	All	1.3	Bores in wood, twigs, or stems	Semisocial	3
	*Tetragonula carbonaria*	Both	All	1.1	Cavity nests	Eusocial	350
Colletidae	*Hylaeus* (*Prosopisteron*) *leai* a	Both	Ap, Sr	1.1	Bores in wood, twigs, or stems	Solitary^b^	3
	*Hylaeus* (*Prosopisteron*) sp. (*undescribed*)	Large	Ap, Sr	0.7	Bores in wood, twigs, or stems	Solitary^b^	2
	*Leioproctus* (*Leioproctus*) sp. (*undescribed*)	Large	Sr	1.7	Soil burrows	Solitary	2
	*Palaeorhiza* (*Cnemidorhiza*) *parallela*	Small	Aa	2.2	Soil burrows	Solitary	1
	*Trichocolletes hackeri*	Large	Ap, Sr	2.5	Soil burrows	Solitary	9
Halictidae	*Homalictus* (*Homalictus*) *atrus* a	Both	Ap, Sr	1.4	Soil burrows	Solitary^b^	43
	*Homalictus* (*Homalictus*) *sphecodoides*	Large	Ap	0.9	Soil burrows	Solitary^b^	1
	*Lasioglossum* (*Chilalictus*) *polygoni*	Both	All	1.7	Soil burrows	Solitary^b^	5
	*Lasioglossum* (*Parasphecodes*) “*BP4*”	Large	Sr	1.6	Soil burrows	Solitary^b^	1
	*Lasioglossum* (*Parasphecodes*) *leichardti*	Both	All	1.7	Soil burrows	Solitary^b^	6
	*Lasioglossum* (*Parasphecodes*) *musicium*	Large	Aa	1.8	Soil burrows	Solitary^b^	8
	*Lasioglossum* (*Parasphecodes*) *sturti*	Both	Ap	1.3	Soil burrows	Solitary^b^	2
	*Lipotriches* (*Lipotriches*) *halictella* a	Large	Aa	1.6	Soil burrows	Solitary^b^	4
	*Mellitidia tomentifera*	Both	Aa, Sr	2.6	Soil burrows	Solitary	50
	*Nomia* (*Paulynomia*) *aurantifer*	Both	Aa, Sr	2.6	Soil burrows	Solitary^b^	3
						Total	607

a The species column indicates species for which identifications are likely, but not certain.

b The sociality column signifies “solitary” species that may share communal nests.

**Table 2:** List of all permutational analyses of variance (PERMANOVA) tests and results in this study, showing factors used and levels of significance

	*df*	SS	MS	Pseudo‐*F*	*p*(mc)	Unique perms
**Multivariate analyses**						
Community composition						
Tree species	2	1,3125	6,562.5	5.20	**0.0001**	9,933
Fragment size	1	8,562.5	8,562.5	6.78	**0.0003**	9,946
Tree sp. × frag. size	2	5,616.5	2,808.2	2.23	**0.0353**	9,940
Fragment ID (tree × frag. size)	15	1,8839	1,255.9	0.98	0.5439	9,903
**Univariate analyses**						
Species richness						
Tree species	2	1.7532	0.8766	1.68	0.2201	9,954
Fragment size	1	2.1465	2.1465	4.10	**0.0563**	9,869
Tree sp. × frag. size	2	0.7188	0.3594	0.69	0.511	9,956
Fragment ID (tree × frag. size)	15	7.8095	0.5206	2.34	0.1775	9,958
Overall abundance						
Tree species	2	19.825	9.9126	2.84	0.0924	9,953
Fragment size	1	72.232	72.232	20.66	**0.0003**	9,870
Tree sp. × frag. size	2	38.955	19.478	5.58	**0.0143**	9,966
Fragment ID (tree × frag. size)	15	52.12	3.4747	1.10	0.4964	9,951
Abundance of *T. carbonaria*						
Tree species	2	61.221	30.61	8.19	**0.0044**	9,941
Fragment size	1	97.504	97.504	26.05	**0.0003**	9,838
Tree sp. × frag. size	2	42.489	21.245	5.69	**0.014**	9,935
Fragment ID (tree × frag. size)	15	55.803	3.7202	0.76	0.6912	9,902
Intertegular distance						
Fragment size	1	85.091	85.091	197.72	**0.001**	305

*Note*

*p*(mc) Values in bold indicate significant effects.

**Figure 5:**
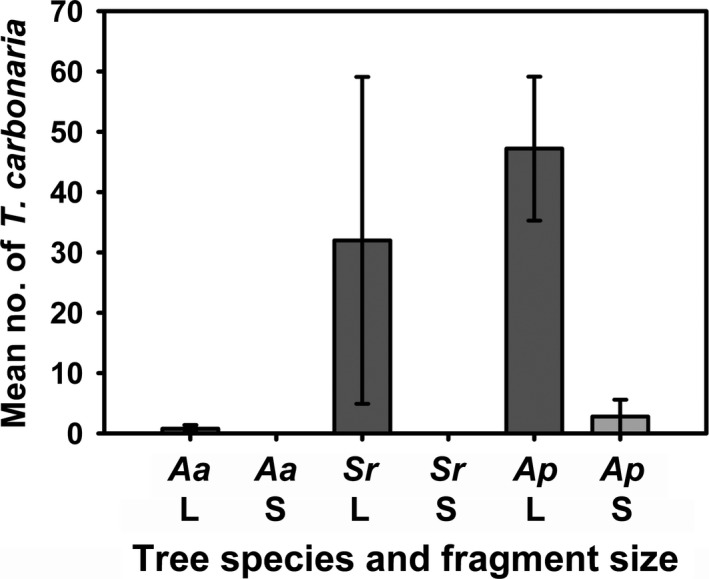
Mean abundance of *Tetragonula carbonaria* among each tree species and fragment size, +/− *SE*. There were no individuals sampled on *Acronichia acidula* or *Sarcopterix reticulata* in small fragments

SIMPER analysis identified four species, *Tetragonula carbonaria*,* Apis mellifera*,* Mellitidia tomentifera*, and *Homalictus (Homalictus) atrus*, that collectively accounted for over 74% of the total community dissimilarity between large and small forest fragments (Table 3). These species were the most frequently collected individuals, collectively totaling 557 of the 607 bees collected in the whole study (Table 1). For *A. acidula* and *S. reticulata* trees, *T. carbonaria* was found only in large fragments, and for *A. petriei*,* T. carbonaria* had lower abundances in small compared to large fragments (Figure 5).

**Table 3:** Similarity percentage analysis (SIMPER) results showing percentage that each of the 10 most influential species contributes to the total dissimilarity in bee assemblages between large and small forest fragments. Individual contribution (%) and cumulative contribution descending (%)

Species	Contrib%	Cum.%
*Tetragonula carbonaria*	32.47	32.47
*Apis mellifera*	21.16	53.63
*Mellitidia tomentifera*	13.17	66.8
*Homalictus* (*Homalictus*) *atrus* a	7.3	74.1
*Trichocolletes hackeri*	4.34	78.44
*Lasioglossum* (*Parasphecodes*) *leichardti*	3.19	81.62
*Lasioglossum* (*Parasphecodes*) *musicium*	3.04	84.66
*Lasioglossum* (*Chilalictus*) *polygoni*	2.7	87.37
*Hylaeus* (*Prosopisteron*) *leai* a	2.36	89.73
*Liptotriches* (*Liptotriches*) *halictella* a	2.12	91.85

*Note*

Average dissimilarity between large and small fragments =79.25%.

a indicates species for which identifications are likely, but not certain.

### 3.2 Functional diversity

**Figure 6:**
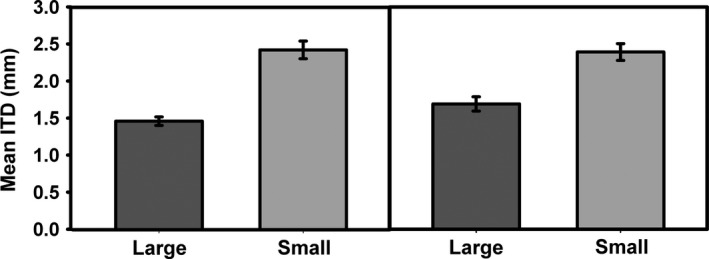
Mean intertegular distance (ITD) of all bees, across all trees, collected in large (dark gray) and small (light gray) fragments. Error bars indicate 95% confidence intervals. Graph on the left shows all bee species, while graph on the right represents data for all species excluding the social bees (*Apis mellifera* and *Tetragonula carbonaria*)

**Figure 7:**
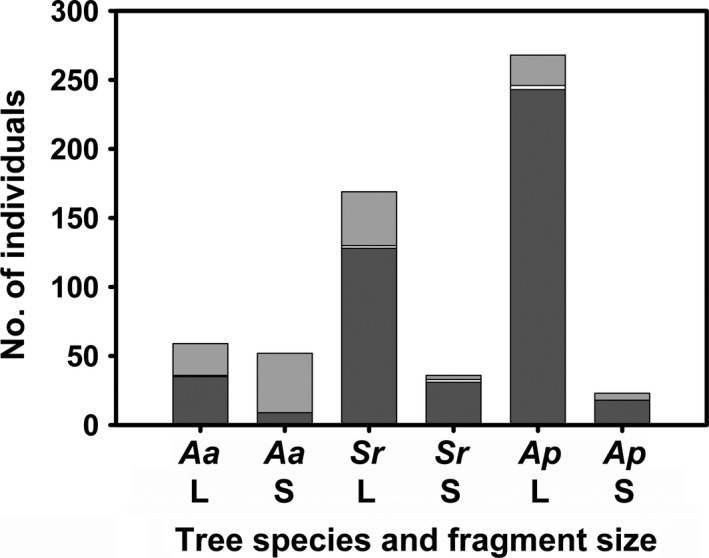
Number of individuals with different nesting strategies across all tree species in each large and small fragments. Dark gray (bottom) = cavity nesters, light gray (top) = soil burrowers, and white (middle) = insect bore, twig, or stem nesters. *Aa *=* Acronichia acidula*;* Sr *= *Sarcopterix reticulata*;* Ap *= *Alphitonia petriei*; L = large fragments; and S = small fragments

The overall mean ITD of bees in large fragments was significantly lower than that in small fragments, and this pattern remained even when both eusocial bee species (*A. mellifera* and *T. carbonaira*) were excluded from the analysis (Figure 6; Table 2). Cavity nesting was the most abundant nesting strategy of bee visitors in all cases except for *A. acidula* growing in small fragments, where soil burrowers were the most abundant visitors (Figure 7).

## 4 DISCUSSION

### 4.1 Small fragments hold functionally dissimilar taxonomic subsets

Ecologists investigating fragmented tropical landscapes are often concerned with the degree to which small fragments support biodiversity and with the ability of these small fragments to continue to maintain important ecosystem functions and services, such as pollination (Hernández‐Ruedas et al., 2014; Turner & Corlett, 1996). In this study, we found significant differences in both the taxonomic and functional diversity of bee assemblages visiting three common native tree species along edges of small and large rainforest fragments. Compared with large fragments, bee assemblages in small fragments were less species rich and abundant, with communities composed of a subset of the species found visiting the same tree species in large forest remnants.

Few studies have investigated the effects of habitat fragmentation on tropical bee visitor assemblages, but studies that do exist have had similar results to ours. Aizen and Feinsinger (1994) found that floral visitor assemblages to neotropic rainforest trees had fewer visits, from a less diverse range of floral visitors, with decreasing size of habitat fragments. Likewise, Olotu, Ndangalasi, and Nyundo (2011) found that the diversity of pollinators to the understory shrub *Mesogyne insignis* was lower in forest fragments than in intact forest in Tanzania. Aguirre and Dirzo (2008) also found reduced abundance of pollinators to a neotropic palm in small forest fragments compared with large fragments. It is important to bear in mind that individuals of the same tree species can vary in attractiveness to pollinators, in part due to both individual tree biology and environmental factors (Frankie et al., 1997). By having adequate replication of trees and remnants, and including multiple tree species, we have reduced the influence of these factors on our results. Future studies will benefit from having greater distances among trees, particularly where there were multiple trees in a single fragment. More importantly, a key component of future work will be to have repeated sampling at individual trees throughout entire flower periods. Individual sampling events for plant's floral visitors can show different diversity patterns through time, and as such, repeated sampling is useful for rigorous floral visitor comparisons among plants (Herrera, 2005; Price et al., 2005). Given that differences in the abundances of just four common species collectively contributed to over 74% of the dissimilarity in community composition between large and small fragments in our study, it is probable that community trends found here are in fact due to changes in the relative abundance of these few bee species.

### 4.2 Reduced abundance of *Tetragonula carbonaria* in small fragments

In our study, we saw a significant reduction in the number of *T. carbonaria* visiting trees in small fragments. *T. carbonaria* is a small‐sized (workers 4 mm long) species of highly eusocial stingless bee (Meliponini), one of the most important tropical bee groups globally. Although little specific information is available about many species in this group, stingless bees are polylectic and are important pollinators of numerous Australian rainforest plants (Williams & Adam, 2010). In addition, stingless bees pollinate a range of tropical and subtropical crop species (Halcroft, Spooner‐Hart, & Dollin, 2013; Heard, 1999, 2016; Slaa, Sanchez Chaves, Malagodi‐Braga, & Hofstede, 2006).

*Tetragonula carbonaria* are cavity‐nesting bees and require tree hollows for nesting. The density of stingless bee nests in rainforest has been positively correlated with the density of large trees (Samejima, Marzuki, Nagamitsu, & Nakasizuka, 2004); however, fragmentation of tropical forests dramatically reduces large tree density (Laurance, Delamonica, Laurance, Vasconcelos, & Lovejoy, 2000), at scales that could be relevant to the differences in area between our large‐ and small‐sized fragments. Unfortunately, we have no data on tree density in our study fragments to investigate this, although this is an important avenue for future research.

*Tetragonula carbonaria* are thought to typically forage at distances of up to 333 m from their nest, with occasional trips up to 400 m (Smith, Heard, Beekman, & Gloag, 2016). This compares well with a similarly sized (<6 mm in length) and related stingless bee species, *Tetragonula minangkabau*, from Indonesia, which has been estimated to forage up to 434 m from the nest (Inoue, Salmah, Abbas, & Yusuf, 1985). Eight of the nine small forest fragments used in our study were <400 m from a nearby larger fragment (Figure 1c), and as such, it is likely that some stingless bees nesting in these large fragments would be capable of flying the distance to the smaller fragments. Yet overall, *T. carbonaria* were almost absent from visitor assemblages in small fragments, suggesting that distance between nesting sites is not the only factor determining stingless bee foraging behavior in these tropical landscapes.

Another factor that may affect stingless bees in countryside landscapes is the amount of nearby forest cover (Brosi et al., 2008). In our study, *T. carbonaria* may potentially be restricted from small forest fragments by their colonial dispersal ability in fragmented rainforest habitats. Stingless bees establish daughter colonies gradually, over weeks or months, with workers travelling from the mother colony each day to build the foundations of the new nest (described in Michener, 2013; van Veen & Sommeijer, 2000). Although no information is available for Australian stingless bees, in other tropical regions, stingless bees have been recorded establishing daughter colonies relatively close to the mother colonies (e.g., at distances lower than their typical foraging ranges) (Inoue, Sakagami, Salmah, & Yamane, 1984; van Veen & Sommeijer, 2000). In Southeast Asian rainforests, stingless bee colonies can be aggregated within individual trees (Eltz, Bruhl, Imiyabir, & Linsenmair, 2003), although in contrast, some neotropic species have uniform densities in rainforest, which may be the result of intraspecific aggression (Hubbell & Johnson, 1977). With these factors in mind, small fragments surrounded by an agricultural matrix devoid of suitable nesting sites (e.g., no large pasture trees) may, to some extent, be isolated from colonial immigration (Brosi, Daily, & Ehrlich, 2007).

Lastly, the reduction of *T. carbonaria* and overall richness and abundance of bees in small fragments in our study could be influenced to some extent by the availability of floral resources and/or interspecific competition between tree species in these fragments. Interspecific competition for pollinators may be higher in species‐rich plant communities such as tropical rainforest (Vamosi et al., 2006), and in areas with reduced pollinator abundance, pollinator visitation to one plant species can be decreased with increases in interspecific floral density (Ye et al., 2014). The population size of the focal plant species may also influence the likelihood of such outcomes (Dauber et al., 2010). It could be that the proximity of *A. petriei* and *S. reticulata* to each other, or to other tree species not included in our study, affect the visitor assemblage to trees of each species, as they flower at the same time. Or simply the floral abundance in our small fragments may be too low to attract high numbers of nonresident bees, or there may be sufficient resources within larger remnants, where nesting occurs, limiting the need to seek resources in small fragments. Future study in this system could incorporate floral diversity and density in fragments too.

Although ours is the first study that reports on Australian stingless bees and the effects of tropical fragmentation, it follows a number of studies from other tropical regions. In a study of tropical forest fragments in Mexico, Calvillo et al. (2010) found no effect of fragment size on the number of stingless bee species present. In fragmented forested landscapes in Costa Rica, Brosi (2009) reported positive relationships between both stingless bee species richness and abundance with increasing forest cover. In contrast, using a landscape genetics approach, Jaffé et al. (2016) demonstrated that in Brazil, the stingless bee *Trigona spinipes* is capable of dispersing among forest fragments across large areas of human‐altered landscapes and even suggest that dispersal of the species may benefit from forest fragmentation and degradation. These studies, and our own, highlight the diverse responses of stingless bee species to habitat fragmentation and also the regional differences found throughout the tropics.

### 4.3 Higher mean ITD in small forest fragments

We found significantly higher mean ITD of bees in small fragments than large, which suggests that there is some functional effect of fragment size on bee assemblages. Size variation among bee species influences how they perform in the environment and how they interact with other species, such as their effectiveness as pollinators to plants with differently structured flowers (Roubik, 1989). As ITD, and general body size, has been positively correlated with flight distance in bee species (Gathmann & Tscharntke, 2002; Greenleaf, Williams, Winfree, & Kremen, 2007; van Veen & Sommeijer, 2000), our results suggest that smaller‐sized bees may be less capable of accessing, or residing in, small fragments than larger‐bodied bees. Our results are consistent with a global meta‐analysis by Williams, Minckley, and Silveria (2001), which found that small‐sized bee species were affected more strongly by habitat isolation than larger‐sized species (although when the authors removed *A. mellifera* from the analysis, there was no longer an effect). It is possible that differences in mean ITD in our study might be a result of the increased isolation of small fragments in the landscape. Recently, in a cross‐continental study, Carrié et al. (2016) found that body size in bee communities was negatively correlated with proximity to seminatural habitat areas, supporting the hypothesis that smaller‐bodied bee species need to nest closer to floral resources because of low dispersal potential (Greenleaf et al., 2007). In addition to floral resources, bees need access to nesting resources such as tree hollows, standing dead wood, and suitable areas of soil. If there are not suitable nesting sites available in, or near to, the small forest fragments in our study, small‐sized species may not be capable of flying the distance to forage in them from elsewhere. Although we did not have enough individuals of each species to investigate this in our study, Renauld, Hutchinson, Leob, Poveda, and Connelly (2016) have demonstrated that increasing land‐use intensification can be associated with reduced body size within individual bee species.

### 4.4 Implications for the provision of pollination as an ecosystem service

While our study focused on grazed landscapes absent of crops and orchards, our results may also have implications for agricultural landscapes that have crops requiring biotic pollination. Due to reduced bee species richness and abundance, small forest fragments may not provide the same pollination services as larger areas of rainforest. Our study joins a growing body of the literature showing reductions in diversity of pollinator communities in smaller rainforest fragments compared with larger areas of forest (e.g., Aizen & Feinsinger, 1994; Calvillo et al., 2010; Smith & Mayfield, 2015). In addition, research from multiple tropical regions has demonstrated declines in crop fruit set with increasing distance from areas of rainforest, due to diminishing floral visits from wild pollinators (Blanche et al., 2006; Ricketts, 2004; Ricketts et al., 2008). Likewise, the diversity of bee visitors in tropical crops can affect the rate of crop pollination, with higher diversity being linked with higher fruit set (Klein, Steffan‐Dewenter, & Tscharntke, 2003). As such, it would seem that landscapes with low densities of small rainforest patches provide poorer pollination services to nearby crops than landscapes with higher densities of large forest patches. In tropical countryside landscapes, small forest fragments are often the most common type of forest remnant. Our results add to the growing literature showing that although such small remnants do have conservation value in tropical countryside landscapes, they do not offer the same benefits to biodiversity or as sources of potential crop pollinators as larger fragments.

## 5 CONCLUSIONS

Our results contribute to the growing body of evidence suggesting that habitat fragmentation can have significant negative effects on bee communities in tropical landscapes. We show that species and functional diversity differ significantly between small and large forest remnants with some important species, including native eusocial stingless bees, being particularly uncommon in small remnants despite the presence of similar floral resources to those sampled in large remnants. Given that small fragments, as defined in this study, are more common than large remnants in many tropical landscapes, our results raise concern over the ongoing functioning of highly fragmented tropical ecosystems in Australia and beyond. Our research suggests the need for larger contiguous areas of forest in tropical countryside landscapes for bee conservation and brings into question the degree to which small rainforest fragments can maintain diverse bee communities and provision important ecosystem services. There are many unmeasured factors that could contribute to our results, and more study is needed to understand all the factors contributing to the loss of certain groups and body sizes from small forest fragments in tropical Australia.

## CONFLICT OF INTEREST

None declared.

## AUTHOR CONTRIBUTIONS

TJS and MMM developed the idea and experimental plan for this experiment together. TJS conducted all field work, laboratory work, analyzed data, and led the writing of the manuscript, with MMM providing substantial feedback and help with manuscript refinement.

## DATA ACCESSIBILITY

All data are available through Dryad https://doi.org/10.5061/dryad.6db84gg.
