# Supplementary material for: The effect of habitat fragmentation on the bee visitor assemblages of three Australian tropical rainforest tree species
Source: Ecol Evol. 2018 Jul 22;8(16):8204–16. doi: 10.1002/ece3.4339 (PMC6144977; doi:10.1002/ece3.4339)

**Supplementary material 1.** List of trees used in this study, and their locations.


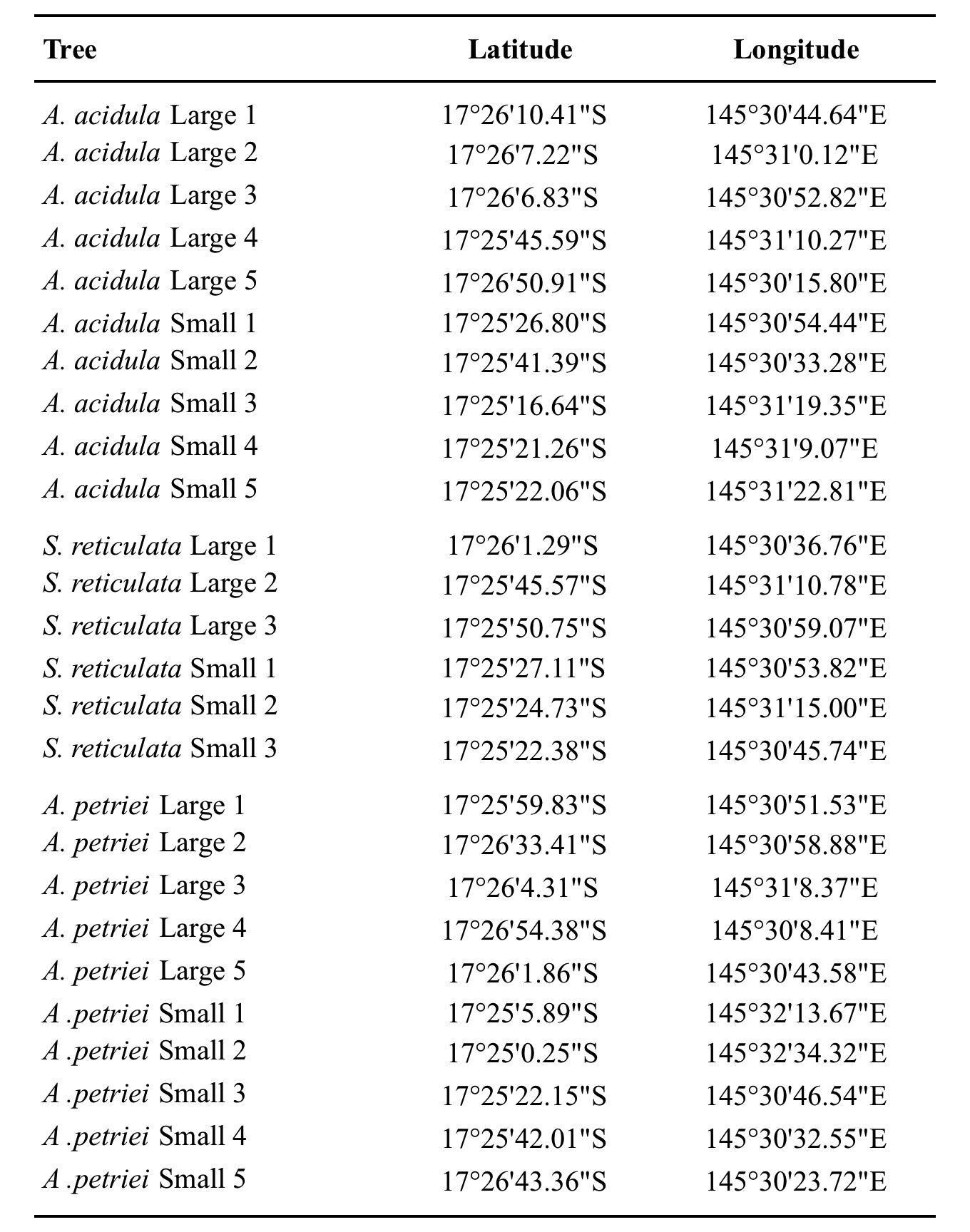


**Supplementary material 2.** Daily temperatures on sampling days in this study. *Sarcopterix reticulata* and *Alphitonia petriei* sampling was conducted during September 2010. During this time maximum and minimum temperatures between 10am and 3pm were recorded on survey days, from a hobo weather data logger housed in a weather station positioned at a central location in the study area (coordinates 342120.26, 8071911.05, GDA94). *Acronichia acidula* was sampled in January and February 2010 when no local weather data logger was operational. For these dates the daily maximum temperatures shown are the whole day maximums, recorded at the closest available Australian Bureau of Meteorology weather station (Walkamin Research Station. Data from: http://www.bom.gov.au/climate/).


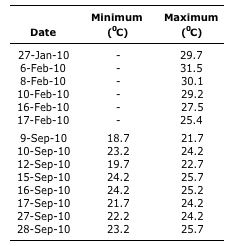


**Supplementary material 3.** Spearman’s Rho correlation values (RELATE analysis), showing no evidence of correlation between the geographic location of trees (latitude and longitude) and any of the taxonomic diversity measures.


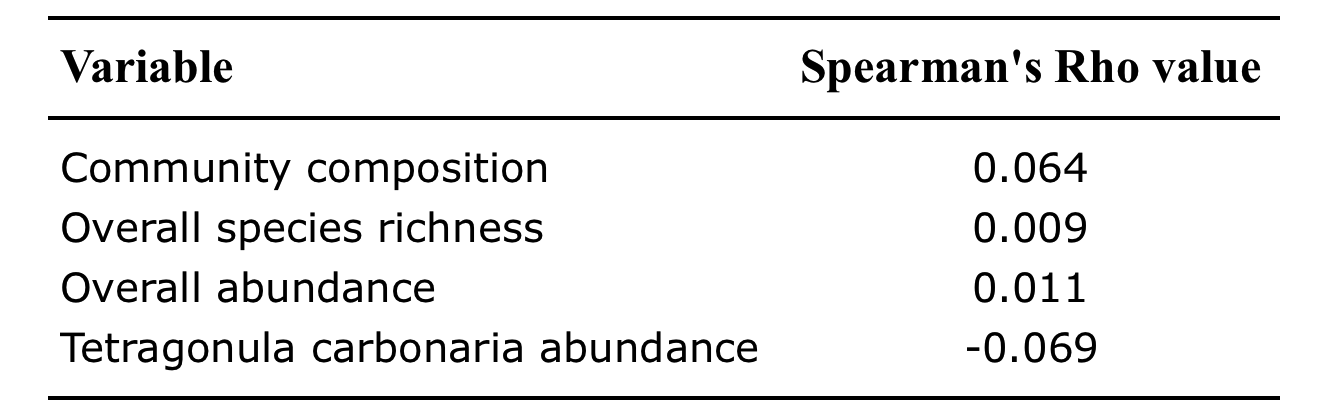

Supplement: Supplementary file 1 [file ECE3-8-8204-s001.docx]
